# A piggybacking mechanism enables peroxisomal localization of the glyoxylate cycle enzyme Mdh2 in yeast

**DOI:** 10.1242/jcs.244376

**Published:** 2020-12-17

**Authors:** Shiran Gabay-Maskit, Luis Daniel Cruz-Zaragoza, Nadav Shai, Miriam Eisenstein, Chen Bibi, Nir Cohen, Tobias Hansen, Eden Yifrach, Nofar Harpaz, Ruth Belostotsky, Wolfgang Schliebs, Maya Schuldiner, Ralf Erdmann, Einat Zalckvar

**Affiliations:** 1 Department of Molecular Genetics, Weizmann Institute of Science, Rehovot 7610001, Israel; 2 Abteilung für Systembiochemie, Institut für Biochemie und Pathobiochemie, Medizinische Fakultät, Ruhr-Universität Bochum, Bochum D-44780, Germany; 3 Division of Pediatric Nephrology, Shaare Zedek Medical Center, Jerusalem, Israel

**Keywords:** Peroxisomes, Protein targeting, Malate dehydrogenase, Glyoxylate cycle, Piggybacking, Pex5

## Abstract

Eukaryotic cells have evolved organelles that allow the compartmentalization and regulation of metabolic processes. Knowledge of molecular mechanisms that allow temporal and spatial organization of enzymes within organelles is therefore crucial for understanding eukaryotic metabolism. Here, we show that the yeast malate dehydrogenase 2 (Mdh2) is dually localized to the cytosol and to peroxisomes and is targeted to peroxisomes via association with Mdh3 and a Pex5-dependent piggybacking mechanism. This dual localization of Mdh2 contributes to our understanding of the glyoxylate cycle and provides a new perspective on compartmentalization of cellular metabolism, which is critical for the perception of metabolic disorders and aging.

## Abstract (precis)

**Highlighted Article:** Yeast Mdh2 is dually localized to the cytosol and peroxisomes, and is targeted to peroxisomes via association with Mdh3 and a Pex5-dependent piggybacking mechanism.

## INTRODUCTION

Eukaryotic cells have evolved organelles that allow the compartmentalization and regulation of multiple parallel metabolic processes requiring unique conditions. One obvious way to control metabolic fluxes is by sequestering enzymes into different compartments, where they would encounter different substrates or co-factors. Indeed, alterations of optimal enzymatic regulation can lead to disease (Metallo and Vander Heiden, 2013). Hence, to understand metabolic disorders, and to enable potential treatments, it is not sufficient to define isolated enzyme functions, but rather it is crucial to investigate the compartmentalization of metabolic pathways and elucidate how enzymes are segregated correctly into organelles.

A central compartment involved in a variety of metabolic pathways in cells is the peroxisome. Indeed, defects in peroxisome functions lead to various metabolic diseases with different severities, such as Zellweger syndrome, Refsum disease, X-linked adrenoleukodystrophy (X-ALD) and primary hyperoxaluria type 1 (Waterham et al., 2016). Although human peroxisomes contain over 50 different enzyme activities (Waterham et al., 2016; Yifrach et al., 2018), our knowledge of the molecular mechanisms that accomplish and regulate the temporal localization of these enzymes and their effect on peroxisome metabolism is still scarce.

In *Saccharomyces cerevisiae* (referred to hereafter as yeast), malate dehydrogenase 2 (Mdh2) is an essential enzyme both for the glyoxylate cycle and for NAD^+^ recycling during β-oxidation of fatty acids (Kunze et al., 2006). Interestingly, both pathways have peroxisomal as well as cytosolic components. However, the cellular localization of Mdh2 is not clear; some reports suggest that Mdh2 is solely localized to the cytosol (Kal et al., 1999; Kunze et al., 2006; McAlister-Henn et al., 1995), whereas other anecdotal observations suggest that Mdh2 is also localized to peroxisomes, but without a clear mechanism of how it could achieve peroxisomal localization (McCammon et al., 1990; Tower et al., 2011). Moreover, the functional consequences of Mdh2 being localized to either the cytosol or peroxisomes, or to both compartments, are not known.

Here, we show that Mdh2 is not only cytosolic but is also localized to peroxisomes. We found that targeting of Mdh2 to peroxisomes requires the formation of a heterocomplex with Mdh3, enabling targeting by a piggyback mechanism via the Pex5 targeting factor. We suggest that the peroxisomal fraction of Mdh2 reduces the need for repeated shuttling of substrates back and forth across the membrane to feed the glyoxylate cycle when the fatty acid oleate is used as the carbon source, while maintaining the flexibility to use Mdh2 in the cytosol during the utilization of ethanol, therefore making it more efficient. More globally, our results demonstrate how targeting by a piggyback mechanism to peroxisomes provides flexibility in enzyme localization.

## RESULTS

### A subpopulation of Mdh2 is localized to peroxisomes

**Fig. 1.**
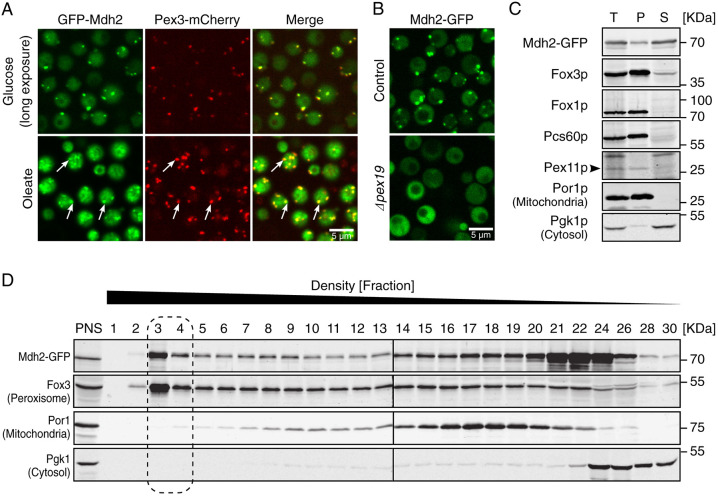
**Mdh2 is localized to the cytosol and to peroxisomes.** (A) When endogenous *GFP–MDH2* is expressed under its native promoter, the GFP–Mdh2 protein is dually localized to the cytosol and to peroxisomes, as seen by colocalization with Pex3–mCherry (in oleate the colocalization is demonstrated by white arrows). Mdh2 expression levels are higher on oleate medium due to the removal of the glucose-induced repression. Therefore, a longer imaging exposure was used for glucose-grown cells. Hundreds of cells were imaged in three technical repeats. (B) Mdh2–GFP (expressed under its native promoter) is no longer localized to puncta in the absence of peroxisomes (Δ*pex19*), proving that the puncta represent peroxisomes. Hundreds of cells were imaged in three technical repeats. (C) Mdh2–GFP (expressed under its native promoter) is present both in the cytosolic fraction (S) and in the organellar pellet (P) of fractionated cells. T, total. Fox3 (also known as Pot1, peroxisomal 3-oxoacyl-CoA thiolase, a PTS2 protein), Fox1 (also known as Pox1, acyl-CoA oxidase), Pcs60 (oxalyl-CoA synthetase, a PTS1 protein) and Pex11 (a peroxisomal membrane protein; arrowhead) were used as peroxisomal markers. Por1 (mitochondrial porin) was used as a mitochondrial marker, and Pgk1 (phosphoglycerate kinase 1) as a cytosolic marker. *n*=2. (D) Density gradient of the postnuclear supernatant (PNS) shows that Mdh2–GFP (expressed under its native promoter) can be found in peroxisomal fractions (marked with a dashed line). Fox3 was used as peroxisomal marker, Por1 was used as a mitochondrial marker and Pgk1 as a cytosolic marker. *n*=2.

To characterize the cellular localization of Mdh2, we tagged the protein at its N terminus with GFP, while not manipulating the native promoter of the gene (Huh et al., 2003; Weill et al., 2018; Yofe et al., 2016), and assayed localization in the presence of either glucose or oleate as a sole carbon source. GFP-tagged Mdh2 was localized to the cytosol but also clearly had a fraction that colocalized with peroxisomes in both glucose- and oleate-grown cells (Fig. 1A). Peroxisomal localization was irrespective of the terminus tagged by GFP, because a C-terminally tagged Mdh2 showed a similar punctate localization pattern (Fig. 1B) that disappeared in the absence of the peroxisome biogenesis factor Pex19, indicative for a redistribution of Mdh2–GFP to a cytosolic-only localization when no peroxisomes are formed (Götte et al., 1998) (Fig. 1B). To verify the microscopic observations, we fractionated yeast cells expressing Mdh2–GFP, which was found in the cytosolic fraction and in a smaller amount in the organelle pellet (Fig. 1C). Mdh2 co-migrated with peroxisomes after isopycnic density centrifugation (Fig. 1D). Hence, a portion of Mdh2 is localized to peroxisomes.

### Peroxisomal Mdh2 is localized to the peroxisome matrix

**Fig. 2.**
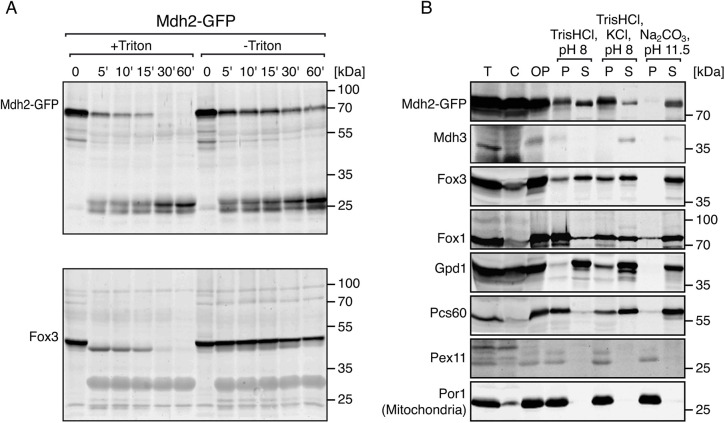
**Mdh2 is localized to the peroxisome matrix.** (A) Protease protection assay of the postnuclear supernatant (PNS) reveals a protease-protected fraction of Mdh2–GFP (expressed under its native promoter). In the presence of Triton X-100, the peroxisomal membrane is permeabilised. Therefore, peroxisomal matrix proteins, such as Fox3, are degraded by proteinase K. Time is indicated in minutes. *n*=2. (B) Chemical extraction of the organellar pellet (OP). A significant proportion of Mdh2–GFP (expressed under its native promoter) remains in the sediment (P, pellet) after low-salt (20 mM TrisHCl, pH 8) treatment of sedimented organelles, suggesting a possible membrane association. Mdh2–GFP is also not extracted by high-salt treatment (20 mM TrisHCl, pH 8 and 500 mM KCl), but is completely extracted to the soluble fraction (S) under treatment with Na_2_CO_3_ pH 11.5, suggesting that the protein is not an integral membrane protein but is peripherally associated with the membrane from the matrix side. T, total; C, cytosolic fraction. Por1 was used as a mitochondrial marker. Por1 was used as a mitochondrial and integral membrane protein marker; Mdh3, Fox3, Fox1, Gpd1 and Pcs60 were used as peroxisomal matrix protein markers with a variable membrane-association propensity; Pex11 was used as a peroxisomal and integral membrane protein marker. *n*=2.

Colocalization of Mdh2 with peroxisomes by microscopy or fractionation experiments cannot differentiate between a peripheral (facing the cytosol) association of Mdh2 or its import into the organelle matrix. To characterize the sub-peroxisomal localization of Mdh2, we applied chemical extractions and protease protection assays to the organellar fraction from strains in which Mdh2 was C-terminally tagged with GFP (Mdh2–GFP). Biochemical analysis revealed a clear protease-protected fraction that became protease-sensitive in the presence of Triton (Fig. 2A), suggesting that the peroxisomal fraction of Mdh2 is not peripheral. Upon chemical extraction of the organellar pellet, Mdh2–GFP was efficiently released from the organelle to the soluble fraction when treated with Na_2_CO_3_, but not when treated with low salt buffer (hypotonic shock) or high salt buffer (500 mM KCl) (Fig. 2B). These results demonstrate that Mdh2–GFP is localized to the peroxisomal matrix and that it might associate with the inner side of the peroxisomal membrane.

### The peroxisomal localization of Mdh2 is Pex5- and Mdh3-dependent

**Fig. 3.**
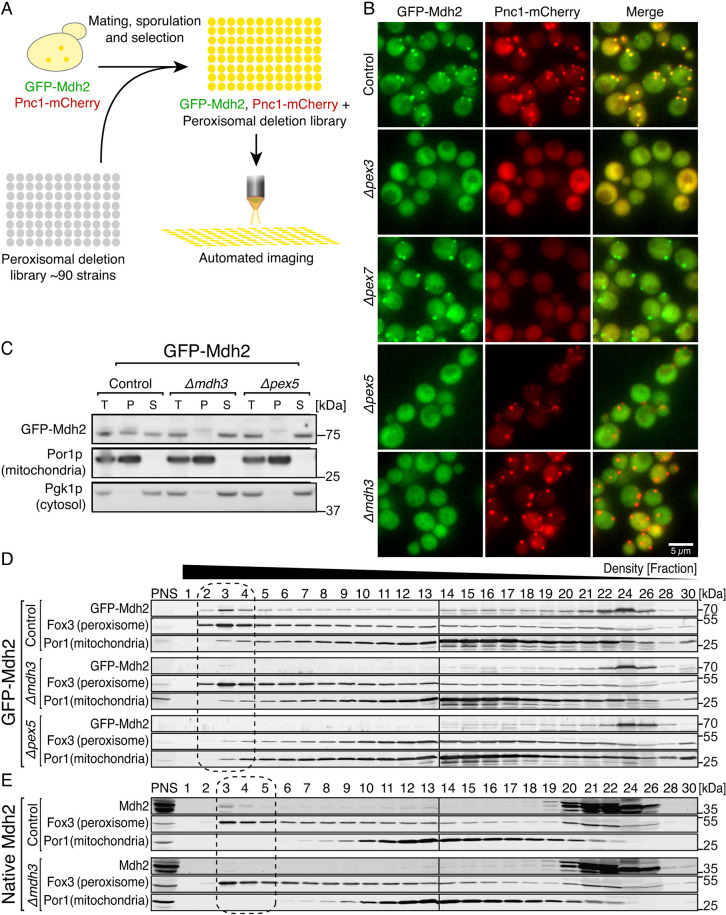
**The peroxisomal localization of Mdh2 is Pex5- and Mdh3-dependent.** (A) Diagram of the high-content screen performed to uncover Mdh2 targeting requirements. Using an automated mating procedure, we created a yeast collection where one peroxisomal gene was deleted in each of the strains, in a background where Mdh2 was N-terminally tagged with GFP and the gene was expressed under a *NOP1* constitutive promoter, and Pnc1 was C-terminally tagged with mCherry (the gene was expressed under its native promoter) to serve as a peroxisomal marker. Using a high-throughput fluorescence microscope, we screened this newly created library searching for strains in which Mdh2 was no longer localized to peroxisomes. (B) GFP–Mdh2 driven by the *NOP1* promoter colocalized with Pnc1–mCherry puncta in control cells, and was localized only to the cytosol in the absence of mature peroxisomes (Δ*pex3*). GFP–Mdh2 was no longer localized to peroxisomes in the absence of Pex5 (Δ*pex5*) or Mdh3 (Δ*mdh3*)*.* Hundreds of cells were imaged in three technical repeats. Pnc1–mCherry was no longer localized to peroxisomes in the absence of Pex7 while GFP–Mdh2 was still localized to puncta (Δpex7). (C) GFP–Mdh2 driven by the *NOP1* promoter is found both in the cytosolic fraction (S) and in the organellar pellet (P) in control cells, but mostly in the cytosolic fraction in the absence of Pex5 and Mdh3. T, total. Por1 was used as a mitochondrial marker and Pgk1 as a cytosolic marker. *n*=4. (D) Density gradient of the postnuclear supernatant (PNS) shows that GFP–Mdh2 driven by the *NOP1* promoter is found in the cytosolic and peroxisomal fractions (marked by a dashed line) in control cells, but is dramatically diminished from the peroxisomal fractions of cells lacking Mdh3 and Pex5. Fox3 is shown as a peroxisome marker and Por1 is shown as a mitochondrial marker. *n*=2. (E) Density gradient of the PNS shows that native Mdh2 is localized to peroxisomal fractions (dashed box) in the control strain but not in Δ*mdh3*. *n*=2.

The localization of Mdh2 to the peroxisome matrix was surprising, because Mdh2 does not contain a canonical peroxisomal targeting signal (PTS) (McAlister-Henn et al., 1995). Hence, we envisioned that Mdh2 has to use a specialized peroxisome targeting machinery. To systematically explore the targeting machinery of Mdh2, we robotically crossed (Cohen and Schuldiner, 2011) a strain in which *GFP–MDH2* was expressed under a strong constitutive promoter (*NOP1pr GFP–MDH2*; Weill et al., 2018; Yofe et al., 2016) with a collection of ∼90 freshly made and verified deletion mutants in peroxisome-related genes (Table S1). The effect of each deletion on GFP–Mdh2 localization was then examined in haploid cells using a high-content imaging platform (Fig. 3A). To exclude general effects of the perturbations on peroxisomes, each strain also contained mCherry-tagged Pnc1, a nicotinamidase known to be imported into peroxisomes by piggybacking on glycerol-3-phosphate dehydrogenase (Gpd1), which carries a type 2 PTS (PTS2) (Effelsberg et al., 2015; Kumar et al., 2016; Saryi et al., 2017). We found that GFP–Mdh2 and Pnc1–mCherry colocalized to punctate structures in the control cells (Fig. 3B, control), whereas they localized to the cytosol when mature peroxisomes were absent (Fig. 3B, Δ*pex3*). Although Pnc1 localization was affected by the absence of the PTS2 targeting machinery, Mdh2 localization was not affected by this perturbation (Fig. 3B, Δ*pex7*). However, GFP–Mdh2 was no longer localized to peroxisomes when the type 1 PTS (PTS1) targeting machinery (Δ*pex5*, or any essential component of the Pex5 import cycle) was absent (Fig. 3B, Δ*pex5* and data not shown). Moreover, Mdh2 was not localized to peroxisomes when *MDH3*, encoding the peroxisomal iso-enzyme of Mdh2, was deleted (Fig. 3B, Δ*mdh3*). No other mutants affected Mdh2 localization. The dependence on Pex5 and Mdh3 was also evident by organelle sedimentation (Fig. 3C) as well as by subcellular fractionations (Fig. 3D).

To ensure that the peroxisomal localization of Mdh2, and its dependence on Mdh3 and Pex5, was not a result of the GFP tagging, we used a specific antibody that recognizes Mdh2 but not the other Mdh isoforms (Gabay-Maskit et al., 2018). Analysis of subcellular fractions clearly demonstrated that native and untagged Mdh2 also co-sediments with peroxisomes (Fig. 3E, control), and that its peroxisomal localization is dependent on the presence of Mdh3 (Fig. 3E, Δ*mdh3*). The observation that Mdh2, a soluble protein that does not contain a canonical PTS, is targeted to peroxisomes in a Pex5- and Mdh3-dependent manner implies that Mdh2 targeting is enabled by binding to, and piggybacking onto, Mdh3, which contains a canonical PTS1.

### Mdh2 binds Mdh3 and is targeted to peroxisomes by piggybacking on Mdh3

**Fig. 4.**
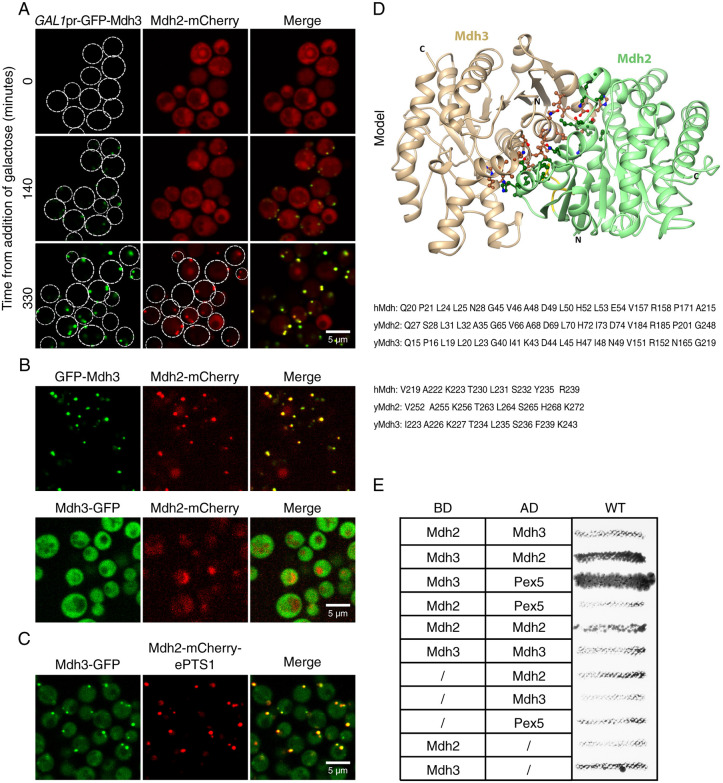
**Mdh2 binds Mdh3, enabling targeting to peroxisomes by a piggyback mechanism.** (A) Mdh2–mCherry (expressed under its native promoter) appeared in puncta only when *GAL1pr*–*GFP–MDH3* was induced (galactose was used as a carbon source), indicating that the peroxisomal localization of Mdh2 is Mdh3-dependent. Hundreds of cells were imaged in three technical repeats. Dashed circles indicate cell outlines. (B) When Mdh3 was N-terminally tagged with GFP and the gene was expressed under control of a *NOP1* constitutive promoter, both Mdh3 and Mdh2 were localized to puncta. However, when Mdh3 was C-terminally tagged with GFP (expressed under its native promoter), and hence the PTS1 was masked, both proteins were solely localized to the cytosol. Hundreds of cells were imaged in three technical repeats. (C) When the PTS1 of Mdh3 (expressed under its native promoter) was masked by GFP and an enhanced PTS1 (ePTS1) was added to Mdh2–mCherry (expressed under its native promoter), both proteins were localized to puncta, implying that a single PTS1 is sufficient to target both Mdh3 and Mdh2 to peroxisomes. Hundreds of cells were imaged in three technical repeats. (D) A predicted structure of the Mdh2–Mdh3 dimer. Mdh2 is shown in green and Mdh3 in beige. The yellow segment in Mdh2 is the insert near the interface. The conserved interface residues are shown as ball and stick, in dark green and brown for the carbon atoms of Mdh2 and Mdh3, respectively, and with oxygen and nitrogen atoms shown in red and blue, respectively. A sequence alignment of the interface residues is shown below the model to indicate the high conservation between human (hMdh) and yeast malate dehydrogenases (yMdh2, yMdh3). (E) Yeast two-hybrid study analyzing the interaction between Mdh2, Mdh3 and Pex5. The results show that Mdh3 but not Mdh2 has the capacity to interact with Pex5 and that Mdh3 and Mdh2 can interact with each other. AD, activating domain; BD, binding domain. *n*=3.

To test the hypothesis that Mdh2 is transported to peroxisomes by piggybacking on Mdh3, we further examined whether the peroxisomal localization of Mdh2 is dependent on Mdh3 expression, and on the ability of Mdh3 to be targeted to peroxisomes. We found that when *GFP–MDH3* was expressed under an inducible *GAL1* promoter (*GAL1pr*–*GFP–MDH3*), Mdh2 only appeared in puncta when the cells were grown on galactose and *MDH3* was expressed (Fig. 4A). Moreover, when we tagged Mdh3 with GFP at the N terminus (leaving the PTS1 exposed), this could confer peroxisomal localization to both Mdh3 and Mdh2 (Fig. 4B, upper panel). However, when the GFP moiety was fused at the C terminus of Mdh3, thus obstructing its PTS1, neither Mdh3 nor Mdh2 could localize to peroxisomes (Fig. 4B, lower panel). This indicates that the PTS1 of Mdh3 is required for targeting of both proteins.

In fact, a single PTS1 was enough to confer targeting of both proteins regardless of which of the two Mdh enzymes carried this signal. When the PTS1 of Mdh3 was masked by a C-terminal GFP tag and a strong PTS1 (DeLoache et al., 2016) was genomically fused to Mdh2 (Mdh2–mCherry–ePTS1), both proteins were localized to peroxisomes (Fig. 4C) and cells could grow on oleate with a small phenotypic growth delay (Fig. S1; compare Mdh3–GFP Mdh2–mCherry–ePTS1, in which Mdh3 piggybacks on Mdh2, to GFP–Mdh3 Mdh2–mCherry, where Mdh2 piggybacks on Mdh3 as in the BY4741 control). The above observations suggest that the proteins form a heterocomplex and hence use a single PTS1 to target to peroxisomes in a Pex5-dependent manner. Additionally, the phenotypic growth delay of the Mdh3–GFP Mdh2–mCherry–ePTS1 strain in both ethanol (EtOH) and oleate implies that a cytosolic pool of Mdh2 is required for an efficient glyoxylate cycle during growth on EtOH as a sole carbon source and for NAD^+^ recycling during β-oxidation of fatty acids during growth on oleate as a sole carbon source.

It has previously been shown that Mdh enzymes can form dimers or tetramers (Goward and Nicholls, 1994; Minárik et al., 2002). Hence, we examined whether heterocomplexes of Mdh2 and Mdh3 can be formed. First, we performed *in silico* structure modeling and found that the dimerization interface shows very high sequence identity between the two yeast Mdh enzymes. This suggests that Mdh2 or Mdh3 homodimers as well as Mdh2–Mdh3 heterodimers are likely to form (Fig. 4D). Notably, the C-termini of the proteins are located away from the dimerization interface, indicating that heterodimerization leaves the PTS1 of Mdh3 exposed. The dimer–dimer interface (tetramerization interface) is smaller than the dimerization interface, yet is highly conserved; therefore, heterotetramers might also form. The modeling predictions were supported by an *in vivo* yeast two-hybrid binding assay that demonstrated that Mdh2 and Mdh3 can interact with each other (Fig. 4E).

To further validate the Pex5-dependent piggybacking mechanism of targeting, we forced Mdh3 to use the PTS2 pathway to enter peroxisomes independently of Pex5. To this end, a PTS2 was fused N-terminally to mCherry–Mdh3 whose PTS1 was abolished (PTS2–mCherry–Mdh3-ΔSKL). Under these conditions, Mdh3 targeting to peroxisomes was less efficient, and PTS2–Mdh3-ΔSKL was localized to the cytosol in addition to puncta (Fig. S2A). Deletion of *PEX7* resulted in the disappearance of the puncta, indicating that the Mdh3 puncta represented peroxisomes and that the targeting was Pex7-dependent, as expected (data not shown). Focusing only on Mdh3 puncta, we could clearly see a reduced capability to efficiently piggyback Mdh2 into peroxisomes (Fig. S2A,B). This result highlights the contribution of Pex5 to the piggyback transport of Mdh2 into peroxisomes. Notably, the ability of Mdh3 to support the targeting of Mdh2 was independent of the catalytic activity of Mdh3, because an inactive mutant of Mdh3 (Mdh3-H187A) could still enable targeting of Mdh2 to peroxisomes (Fig. S2A,B). Taken together, our results suggest that Mdh2 and Mdh3 form a heteromeric complex that enables targeting of Mdh2, which lacks a PTS, to peroxisomes by piggybacking. This is the first example of Pex5-dependent piggybacking of a natural, non PTS1-containing protein in yeast, and it changes our view on the spatial distribution of Mdh2.

## DISCUSSION

In this work we demonstrate that Mdh2, a non-PTS protein, previously believed to be exclusively localized in the cytosol, is also localized to peroxisomes. We show that the peroxisomal localization of Mdh2 is mediated by piggybacking on Mdh3 in a Pex5-dependent manner. Although it has been demonstrated that PTS1-based piggybacking is possible in *S. cerevisiae* (McNew and Goodman, 1994; Yang et al., 2001), we here show the first example of a natural, non PTS1-containing protein that is targeted to peroxisomes by piggybacking on a PTS1 protein in this yeast. Our observations outline a mechanism of piggyback import to peroxisomes that is factor specific (Pex5) and is different from the Pex7-dependent Pnc1–Gpd1 import (Effelsberg et al., 2015; Kumar et al., 2016; Saryi et al., 2017). Despite their co-import, Mdh3 and Mdh2 behaved differently in chemical extraction experiments (Fig. 2B). This raises the possibility that the two Mdh proteins dissociate following translocation into peroxisomes. Alternatively, this could reflect an organization of a salt-sensitive heterocomplex in which Mdh2 is tightly associated with the inner side of the peroxisomal membrane and Mdh3 faces the matrix.

What could be the cellular benefit of having such a dual carbon-source-dependent localization of Mdh2? Mdh2 is part of the glyoxylate cycle that enables the conversion of acetyl-CoA into succinate. This cycle is essential during growth in the absence of glucose, when simple carbon sources must be utilized for anabolic reactions. Upon growth on oleate, acetyl-CoA is produced in the peroxisomal matrix as a major product of peroxisomal fatty acid oxidation, whereas upon growth in EtOH, acetyl-CoA is produced in the cytosol. Originally, it was hypothesized that the acetyl-CoA produced inside peroxisomes during growth in fatty acid-containing medium needs to be exported from peroxisomes. Then, it can be further metabolized in mitochondria or processed by the cytosolic glyoxylate cycle.

However, the placement of Mdh2 in the peroxisomal matrix during growth on oleate and in the cytosol upon growth on EtOH brings the enzymes closest to the source of their most valuable substrate, and thus optimizes the glyoxylate cycle according to the cellular needs.

**Fig. 5.**
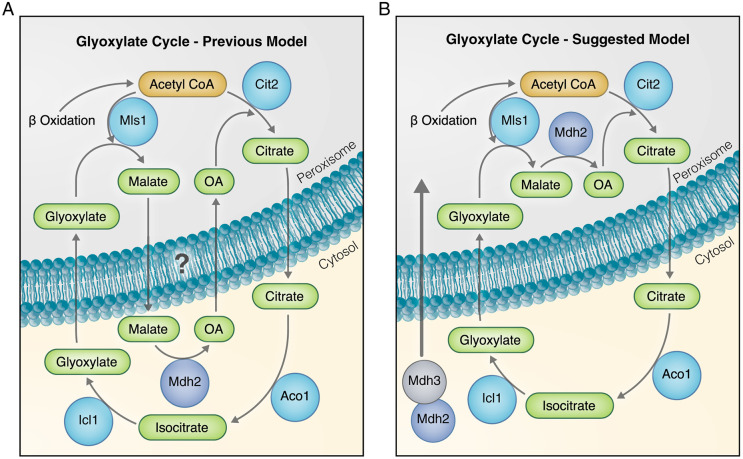
**Mdh2 localization to peroxisomes can contribute to the efficiency of the glyoxylate cycle.** (A) A graphical model of the previous hypothesis on Mdh2 localization. Formerly, Mdh2 was proposed to be exclusively localized to the cytosol, where it plays a role in the glyoxylate cycle. (B) We suggest a new working model that takes into account our new findings that Mdh2 is also targeted to peroxisomes by piggybacking on Mdh3. The peroxisomal localization of a pool of Mdh2 increases the efficiency of the glyoxylate cycle during growth in fatty acids, because three consecutive reactions of the glyoxylate cycle can occur inside peroxisomes (catalyzed by Mls1, Mdh2 and Cit2), thereby eliminating the need to transport malate out of peroxisomes and oxaloacetate into peroxisomes during the glyoxylate cycle. OA, oxaloacetate.

In line with the idea that Mdh2 localization to peroxisomes supports optimal glyoxylate cycle function when cells consume oleate, it has been shown that two additional enzymes of the glyoxylate cycle, malate synthase 1 (Mls1, which converts acetyl-CoA and glyoxylate into malate) and citrate synthase 2 (Cit2, which converts acetyl-CoA and oxaloacetate to citrate), are localized in peroxisomes upon growth on oleate (Kunze et al., 2006). However, because Mdh2 was previously thought to be only cytosolic, it has been proposed that under these conditions, there is ‘back and forth’ shuttling of metabolites across the peroxisomal membrane to interface with the enzymes that remain cytosolic [aconitase 1 (Aco1), isocitrate lyase 1 (Icl1) and Mdh2; Kunze et al., 2006] (Fig. 5A). Our data suggest that the metabolic wiring is, in fact, much simpler (Fig. 5B) – the peroxisomal localization of a pool of Mdh2 could increase the efficiency of the glyoxylate cycle during growth in fatty acids, because three consecutive reactions of the glyoxylate cycle could occur inside peroxisomes (catalyzed by Mls1, Mdh2 and Cit2), thereby eliminating the need to transport malate out of, and oxaloacetate into, peroxisomes (Fig. 5). Conversely, when cells grow on EtOH, and acetyl-CoA is available in the cytosol, it would be beneficial for Mdh2 and the other enzymes that are part of the glyoxylate cycle to localize to the cytosol. Indeed, previous observations demonstrate that Mls1 is localized mostly to the cytosol when cells consume EtOH, and is only present in peroxisomes when cells consume fats (Kunze et al., 2006). Along these lines, it has recently been shown that Mls1 targeting can be mediated by Pex9, a factor that targets a specific subset of PTS1 proteins into peroxisomes when it is induced in oleate (Effelsberg et al., 2016; Yifrach et al., 2016).

In support of this metabolic logic, our data show that when we force Mdh2 into peroxisomes using a strong PTS1, the capacity of yeast to grow on EtOH is dramatically reduced (Fig. S1, compare the growth on EtOH between Mdh3–GFP Mdh2–mCherry–ePTS1, in which most Mdh2 is in peroxisomes, to GFP–Mdh3 Mdh2–mCherry, where all Mdh2 is cytosolic). Hence, dynamic shuttling of Mdh2 between compartments is essential for efficient regulation of the glyoxylate pathway in different metabolic conditions. Piggybacking of Mdh2 on Mdh3 can serve as a dynamic posttranslational regulatory mechanism to control the peroxisomal pool of Mdh2 under different metabolic conditions and enable an efficient metabolic flux upon cell demand (Kabran et al., 2012).

Notably, the activity of Mdh2 is also essential for maintaining NAD^+^ levels required for continuous β-oxidation of fatty acids (Kunze et al., 2006). It is unclear whether the presence of Mdh2 in peroxisomes during growth in oleate may also contribute to this activity. It has previously been shown (Steffan and McAlister-Henn, 1992) that the *K_m_* value of Mdh2 for oxaloacetate is lower than the *K_m_* of Mdh3 (Mdh2=0.07 mM, Mdh3=0.3 mM). Hence, under the conditions used, the affinity of Mdh2 to oxaloacetate is higher than that of Mdh3. This implies that, in fact, Mdh2 should be more efficient in the conversion of oxaloacetate to malate – a process required for NAD^+^ replenishing. Thus, Mdh2 that is targeted to peroxisomes by piggybacking on Mdh3 could enhance the conversion of oxalacetate to malate, optimizing β-oxidation.

Interestingly, in mammals, where NAD^+^ recycling is suggested to be mediated by lactate and pyruvate and not by malate–oxaloacetate (Baumgart et al., 1996; Wanders et al., 2015), it has been shown that lactate dehydrogenase B (LDHB) can acquire a PTS1 sequence by translational read-through. Only when the PTS1 of LDHB is exposed, and hence LDHB is targeted to peroxisomes, can LDHA, a heteromeric subunit of the LDH enzyme, also be localized to peroxisomes (Schueren et al., 2014). This suggests that the concept of piggybacking of coupled enzymes that are required for NAD^+^ recycling in peroxisomes is evolutionarily conserved and strengthens our hypothesis that this piggybacking has a functional role. In addition, it has been shown that human MDH1 can undergo a cell-specific read-through and, as a result, acquire a PTS1 (Hofhuis et al., 2016). This shows that malate dehydrogenases can harness different mechanisms to be targeted to peroxisomes. Taken together, this suggests that different levels of regulation, including piggybacking, control the dynamic localization of peroxisomal enzymes under different metabolic conditions. Such targeting may very well exist for other peroxisomal enzymes. Understanding spatial localization of enzymes at the cellular level will enable us not only to better understand how the basic unit of life, the cell, works under different metabolic needs, but will create insights into the nature of certain metabolic disorders.

## MATERIALS AND METHODS

### Yeast strains and strain construction

All strains in this study are based on the BY4741 laboratory strain (Brachmann et al., 1998). A complete list of strains can be found in Table S2. The strain expressing GFP–Mdh2 under its native promoter (Fig. 1A) was picked from the seamless N-terminal GFP library (Yofe et al., 2016). The strain PJ69-4A wild type (James et al., 1996) was used for yeast two-hybrid studies.

### Peroxisome deletion library

Knockouts were established by homologous recombination using a kanamycin selection cassette that was amplified from a pFA6a-KanMX6 plasmid (pMS47 in Table S3). Primers to amplify the selection cassette from a plasmid with flanking sequences directing it to recombine in place of the target gene were designed using the Primers-4-Yeast web tool (Yofe and Schuldiner, 2014; http://wws.weizmann.ac.il/Primers-4-Yeast) using the ‘KO pFA6’ option. All primers included a 40 bp homology sequence followed by 20 bp of cassette amplification sequence. The homology sequences were upstream of the protein start codon and downstream of the stop codon, as described in the Primers-4-Yeast web tool. Primers for validation of the deletion transformations were also designed with the Primers-4-Yeast web tool, using the appropriate ‘W.T CHK’ option. Primers were manufactured by Sigma-Aldrich in 96-well plates.

### Plasmids and primers

All plasmids used in this study can be found in Table S3. All primers used in this study can be found in Table S4.

#### pFA6a-mCherry-ePTS1-HIS3MX6

Generation of pFA6a-mCherry-ePTS1 was performed by whole plasmid amplification followed by ligation of the linear PCR product. Plasmid pMS623 pFA6a-mCherry-ΔSKL was used as a template for amplification with the following primers: mCherry_SKL_F (5′-TCCAAATTGTAAGCGAATTTCTTATGATTTATG-3′) and mCherry_SKL_R (5′-TCTTCTACCTCTTCCCAATGAACCCTTGTACAGCTCGTCCATGCC-3′). This plasmid was used to prepare the Mdh2–mCherry–ePTS1 strain. The sequence of the ePTS1 signal (GSLGRGRRSKL) was adopted from DeLoache et al. (2016).

#### Yeast two-hybrid plasmids

For the preparation of pLDC31, the *MDH2* gene was amplified from yeast genomic DNA using the primers RE5675 and RE5676 (Table S4). The PCR product and the vector pPC86 were digested with *Sal*I and *Not*I endonucleases and ligated. The plasmid pLDC32 was prepared by isolating the *MDH2* segment from pLDC31 *Sal*I/*Not*I digested, and ligating it into *Sal*I/*Not*I digested pPC97. The plasmids pPC86-Mdh3 and pPC97-Mdh3 were provided by Anirban Chakraborty (Department of System Biochemistry, Ruhr-University Bochum, Germany).

### Antibodies

For immunodetection, the previously described antibodies were used: anti-GFP–GST (1:10,000; Birschmann et al., 2003), anti-Mdh3 (1:10,000; Steffan and McAlister-Henn, 1992), anti-Fox1 (1:10,000; Schäfer et al., 2004), anti-Fox3 (1:10,000; Erdmann and Kunau, 1994), anti-Gpd1 (1:10,000; Effelsberg et al., 2015), anti-Pcs60 (1:10,000; Blobel and Erdmann, 1996), anti-Pex11 (1:10,000; Erdmann and Blobel, 1995), anti-Pgk1 (1:7000; ThermoFisher Scientific Cat. # 459250.) and anti-Por1 (1:10,000; Kerssen et al., 2006). The Mdh2 antibody was produced by GeneScript, USA, using a specific Mdh2 peptide as an immunogen (MPHSVTPSIEQDSLC), and was previously verified by us for specificity and activity (1:2500; Gabay-Maskit et al., 2018). The immunodecorated membranes were incubated with IRDye 800CW goat anti-rabbit IgG or IRDye 800CW goat anti-mouse IgG (LI-COR Bioscience) as secondary antibodies, and detected with an Odyssey Infrared imaging system (LI-COR Bioscience).

### Yeast library preparation

We created a yeast collection of haploid strains containing *NOP1*pr-GFP–Mdh2, a peroxisomal marker (Pnc1–mCherry) and a deletion in one peroxisomal gene (Fig. 3A). The query strain was constructed on the basis of an automated mating compatible strain (for further information see Table S2). Using an automated mating method (Cohen and Schuldiner, 2011; Yan Tong and Boone, 2005) the GFP–Mdh2, Pnc1–mCherry query strain was crossed with the peroxisome deletion library. To perform the manipulations in high-density format we used a RoToR benchtop colony arrayer (Singer Instruments). In brief, mating was performed on rich medium plates [Agar, YPD (Formedium)], and selection for diploid cells was performed on SD −URA −HIS plates (Agar, 2% glucose, 0.67% YNB, amino acid mixture) containing Geneticin (200 μg/ml) and/or Nourseothricin (200 μg/ml). Sporulation was induced by transferring cells to nitrogen starvation medium plates [Agar, amino acid mix -uracil -tryptophan, 0.05 mM uracil, 0.05 mM tryptophan, potassium acetate (10g/ml)] for 7 d. Haploid cells containing the desired mutations were selected by transferring cell to SD −URA −HIS plates plates containing Geneticin (same concentration as above), alongside the toxic amino acid derivatives canavanine (50 mg/l) and thialysine (50 mg/l) (Sigma-Aldrich) to select against remaining diploids, and lacking leucine to select for spores with an α mating type.

### Automated high-throughput fluorescence microscopy

The yeast collections were visualized using an automated microscopy setup, as described previously (Breker et al., 2013). In brief, cells were transferred from agar plates into 384-well polystyrene plates (Greiner) for growth in liquid medium using the RoToR arrayer robot (Singer Instruments). Liquid cultures were grown in a LiCONiC incubator, overnight at 30°C in SD −URA −HIS medium. A JANUS liquid handler (PerkinElmer) connected to the incubator was used to dilute the strains to an OD_600_ of ∼0.2 into plates containing SD medium (6.7 g/l yeast nitrogen base and 2% glucose) supplemented with complete amino acids. Plates were incubated at 30°C for 4 h. The cultures in the plates were then transferred by the liquid handler into glass-bottom 384-well microscope plates (Matrical Bioscience) coated with concanavalin A (Sigma-Aldrich). After 20 min, cells were washed three times with SD-riboflavin complete medium to remove non-adherent cells and to obtain a cell monolayer. The plates were then transferred to the ScanR automated inverted fluorescence microscope system (Olympus) using a robotic swap arm (Hamilton). Images of cells in the 384-well plates were recorded in the same liquid as the washing step at 24°C using a 60× air lens (NA 0.9) and with an ORCA-ER charge-coupled device camera (Hamamatsu). Images were acquired in two channels: GFP (excitation filter 490/20 nm, emission filter 535/50 nm) and mCherry (excitation filter 572/35 nm, emission filter 632/60 nm). All images were taken at a single focal plane.

### Manual microscopy

For manual microscopy, yeast strains were grown as described above for the high-throughput microscopy, except that after the overnight growth, strains were diluted to SD medium or S-Oleate (6.7 g/l yeast nitrogen base. 0.2% oleic acid and 0.1% Tween-80), all with complete amino acids. Strains were then incubated at 30°C for 4 h (SD) or 20 h (S-Oleate medium). Images of cells in the 384-well plates were recorded in the same growth medium or in double-distilled water for S-Oleate-growing cells. Imaging was performed using the VisiScope Confocal Cell Explorer system, composed of a Zeiss Yokogawa spinning disk scanning unit (CSU-W1) coupled with an inverted Olympus microscope (IX83; 60× oil objective; excitation wavelength of 488 nm for GFP and 501 nm for mCherry). Images were taken using a connected PCO-Edge sCMOS camera controlled by VisView software. All images were taken at a single focal plane.

### Cellular fractionation

For cell fractionation experiments, yeast cells were first cultured on YNBG medium [1 g/l yeast extract, 1.7 g/l yeast nitrogen base without amino acids (YNB), 5 g/l ammonium sulfate and 3 g/l glucose, pH 6] for 8 h. Peroxisome proliferation was induced by addition of 5× Oleate-YNB medium [1 g/l yeast extract, 1.7 g/l yeast nitrogen base without amino acids (YNB), 5 g/l ammonium sulfate, 0.5% (v/v) Oleic acid and 0.25% (v/v) Tween-40, pH 6.0]. The culture was further incubated for 15 h. The cells were harvested and processed for postnuclear supernatant preparation (PNS), performed as described previously (Cramer et al., 2015). The PNS (10–15 mg of protein) was loaded on top of a 2–36% (w/v) OptiPrep/iodixanol linear gradient, containing 18% (w/v) sucrose in 35-ml ultracrimp tubes (Thermo Scientific). The tubes were centrifuged in the vertical rotor TV-860 (19,500 rpm, 36,000 ***g***; 1 h 45 min, 4°C) (Cramer et al., 2015). Finally, the gradient was separated into thirty fractions. Equivalent volumes were treated with trichloroacetic acid (TCA), separated by SDS-PAGE and analyzed by immunodetection.

For the complete sedimentation of cellular organelles, including peroxisomes, 500 µl of PNS, with a protein concentration of 2 mg/ml, was loaded onto a 500 µl cushion of 5% (w/v) sucrose in lysis buffer (5 mM MES, 0.5 mM EDTA, 1 mM KCl and 0.6 M sorbitol, pH 6.0) and centrifuged (20,000 ***g***, 40 min, 4°C; MLA-130 rotor). The organellar pellet was resuspended in lysis buffer. Equivalent fraction volumes were treated with TCA, separated by SDS-PAGE and analyzed by immunodetection.

### Subperoxisomal protein localization studies

The subperoxisomal distribution of Mdh2 and other proteins was assessed using chemical protein extraction from sedimented organelles. Briefly, the crude organellar pellet was prepared by centrifugation of the PNS. Two cushions were applied in the tube: the first one consisted of 1 ml of 60% (w/v) sucrose in lysis buffer, and the second of 1 ml of 5% (w/v) sucrose in lysis buffer. Next, 8 ml of PNS (2 mg/ml protein concentration) was loaded onto the cushions and centrifuged (21,600 rpm, 80,000 ***g***; 44 min, 4°C) in a SW41 rotor (Beckman Coulter). The supernatant was removed, and the pellet was resuspended in 8 ml of cold lysis buffer. Subsequently, 500 µl of the suspension was applied onto a cushion of 7% (w/v) sucrose in lysis buffer (three samples per strain were prepared). The organelles were sedimented by centrifuging at 40,000 ***g*** for 21 min at 4°C in an MLA-130 rotor (Beckman Coulter). The supernatant was removed, and the pellet resuspended in equal volumes of different buffers: (1) Tris pH 8.0 (low salt buffer; 10 mM Tris-HCl, pH 8.0); (2) Tris-KCl pH 8.0 (high-salt buffer; 10 mM Tris-HCl and 500 mM KCl, pH 8.0); and (3) carbonate pH 11.5 (100 mM Na_2_CO_3_/NaHCO_3_, pH 11.5). The suspensions were incubated for 1 h at 4°C on a rotating wheel. Afterwards, the membranes were sedimented through a cushion of 7% (w/v) sucrose in the corresponding buffer at 200,000 ***g***, 40 min, 4°C in an MLA-130 rotor (Beckman Coulter). The supernatant was transferred to a new tube, and the same treatment was repeated with the sediment obtained. Both supernatants were mixed. The pellet was resuspended in 500 µl of lysis buffer. All samples were precipitated with TCA, and equivalent volumes were analyzed by immunodetection.

For the protease protection experiments, PNSs were prepared in lysis buffer with an incomplete inhibitor mixture (only NaF, Bestatin and Pepstatin). First, 30 µl of 20% (v/v) Triton X-100 was added to 3 ml of PNS (protein concentration 1 mg/ml). A sample of 450 µl was taken (T0). Then, 50 µl of 1 mg/ml Proteinase K in lysis buffer was added to each mixture. Subsequently, a sample was taken after 5, 10, 15, 30 and 60 min of incubation on ice, and the proteolysis was stopped by adding 10 µl of 0.2 M PMSF. The proteins were precipitated by TCA treatment, separated by SDS-PAGE and analyzed by immunodetection.

### Yeast two-hybrid analysis

The *S. cerevisiae* strain PJ69-4A was employed for the yeast two-hybrid experiments. Two plasmids derived from pPC86 (*GAL4*pr-activation domain, AD) and pPC97 (*GAL4*p-binding domain, BD) (Chevray and Nathans, 1992) were co-transformed into the cells, in different combinations (James et al., 1996). The clones containing both plasmids were selected on 2% Glu-YNB –Leu −Trp agar plates [0.1 % (w/v) yeast extract, 0.17 % (w/v) YNB (yeast nitrogen base without amino acids), 0.5 % (w/v) (NH_4_)_2_SO_4_, amino acid mix for selection, 2% (w/v) glucose, 2% (w/v) agar, pH 6.0].

Three clones from each combination were transferred onto a 2% Glu-YNB –Leu −Trp agar plate. The plate was incubated for 2 d and then replicated by stamping with sterile cloth, on plates containing 2% Glu-YNB –Leu −Trp, or 2% Glu-YNB –Leu −Trp –His +5 mM 3-amino-1,2,4-triazole (3-AT). The plates were incubated for 3–6 d. Longer incubation time was required in order to detect very weak interactions.

### Molecular modeling

Model structures of yeast Mdh2 and Mdh3 were constructed by comparative modeling, using the program Modeller (Šali and Blundell, 1993) as implemented in UCSF-Chimera (Pettersen et al., 2004). The modeling templates for Mdh2 and Mdh3 were human Mdh type 2 (MDH2), porcine mitochondrial MDH2 and glyoxysomal Mdh2 from *Citrullus lanatus* [Protein Data Bank (PDB) IDs 2DFD, 1MLD and 1SMK, respectively]. The sequence identity to the three templates was 46–47% for yeast Mdh2 and 47–48% for yeast Mdh3. Two of the templates, 2DFD and 1MLD, are homotetramers and the third is a homodimer, but the large dimerization interface is conserved and very similar in the three templates and in other Mdh structures.

A model of the (Mdh2–Mdh3)_2_ heterotetramer was constructed by superposing two Mdh2–Mdh3 dimers onto the tetrameric structure 2DFD. This starting structure was placed in a box of water, the system was neutralized and energy minimized, followed with a molecular dynamics simulation using Gromacs (Van Der Spoel et al., 2005), first 0.5 ns solvent equilibration and then 5 ns simulation of the whole system. In the last step, the Cα atoms of the helical segments were restrained to their starting positions, whereas segments with inserts or deletions compared to the modeling templates, and all the loops, were free to move. UCSF-chimera was used for visualization of the docking results and preparation of Fig. 4D.

### Growth assays

For the growth assays on different carbon sources (Fig. S1), strains were grown overnight at 30°C in synthetic medium (6.7 g/l yeast nitrogen base) with 0.1% glucose (low glucose SD), supplemented with complete amino acids. The cells were then diluted and incubated at 30°C for 6 h in low glucose SD to reach an OD_600_ of ∼0.6. Cells were then washed with double-distilled water, and OD_600_ was confirmed after the wash. Cells were then diluted to an OD_600_=0.1, followed by three additional tenfold serial dilutions. Then, 2.5 µl of each dilution was placed on SD, SEtOH (6.7 g/l yeast nitrogen base and 2% ethanol) and SOleate (6.7 g/l yeast nitrogen base, 0.2% oleic acid and 0.1% Tween-80) plates, all with complete amino acids. The plates were incubated for 2 d (SD), 11–15 d (SEtOH) or 6–8 d (SOleate) at 30°C, and then imaged using a Nikon Coolpix P510 camera.

### Mdh3 targeting through the PTS2 pathway

A cassette containing the promoter/terminator pair *MET25pr-ADHt* was inserted between the restriction sites *Xho*I/*Bam*HI in the vector pRS415 to generate pLDC33. Afterwards, the gene coding for mCherry was amplified using the primers RE5685 and RE5686 (Table S4). Alternatively, *mCherry* was amplified in two PCR steps, first with the primers RE5687 and RE5686, and then the product was amplified with primers RE5688 and RE5686, to incorporate the PTS2 signal from Fox3 (*Sc*Fox3_1–15_) at the N-terminus of Cherry (PTS2–mCherry). Both mCherry and PTS2–mCherry were inserted into pLDC33 between the *Sal*I/*Bgl*II restriction sites, leading to pLDC34 and pLDC35, respectively.

In the final step, full-length Mdh3 (*MDH3*) or a truncated version without the PTS1 signal (*MDH3ΔSKL*) were amplified from genomic DNA with the primer pairs RE5689/RE5690 and RE5689/RE5691, respectively. The *MDH3* PCR product was inserted into pLDC34, between the *Mlu*I/*Bgl*II restriction sites, generating the plasmid pLDC36. The *MDH3ΔSKL* PCR product was inserted in the same manner into pLDC35, resulting in pLDC39. Additionally, the mutation *H187A* was introduced into *MDH3* gene (in pLDC36) via site-directed mutagenesis using the primers RE6125 and RE6126, obtaining pLDC53. The yeast genomic DNA was isolated from wild-type cells using Harju's method (Harju et al., 2004).

### Image analysis for quantifying Mdh2–GFP colocalization with mCherry–Mdh3 constructs

To quantify colocalization, Δ*mdh3*; Mdh2–GFP strains expressing mCherry–Mdh3, PTS2–mCherry–Mdh3-ΔSKL or mCherry–Mdh3-H187A were grown in synthetic medium without methionine and were imaged using spinning-disk microscopy. For each experiment, 100 mCherry–dh3 puncta were manually scored for colocalization with Mdh2–GFP. *n*=2 biological repeats, 200 puncta per strain.

## Supplementary Material

Supplementary information

## References

[JCS244376C1] Baumgart E., Fahimi H. D., Stich A. and Volkl A. (1996). L-lactate dehydrogenase A_4_- and A_3_B isoforms are bona fide peroxisomal enzymes in rat liver. Evidence for involvement in intraperoxisomal NADH reoxidation. *J. Biol. Chem.* 271, 3846-3855. 10.1074/jbc.271.7.3846 8632003

[JCS244376C2] Birschmann I., Stroobants A. K., van den Berg M., Schafer A., Rosenkranz K., Kunau W. H. and Tabak H. F. (2003). Pex15p of Saccharomyces cerevisiae provides a molecular basis for recruitment of the AAA peroxin Pex6p to peroxisomal membranes. *Mol. Biol. Cell* 14, 2226-2236. 10.1091/mbc.e02-11-0752 12808025 PMC194873

[JCS244376C3] Blobel F. and Erdmann R. (1996). Identification of a yeast peroxisomal member of the family of AMP-binding proteins. *Eur. J. Biochem.* 240, 468-476. 10.1111/j.1432-1033.1996.0468h.x 8841414

[JCS244376C4] Brachmann C. B., Davies A., Cost G. J., Caputo E., Li J., Hieter P. and Boeke J. D. (1998). Designer Deletion Strains derived from Saccharomyces cerevisiae S288C: a Useful set of Strains and Plasmids for PCR-mediated Gene Disruption and Other Applications. *Yeast* 14, 115-132. 10.1002/(SICI)1097-0061(19980130)14:2<115::AID-YEA204>3.0.CO;2-2 9483801

[JCS244376C5] Breker M., Gymrek M. and Schuldiner M. (2013). A novel single-cell screening platform reveals proteome plasticity during yeast stress responses. *J. Cell Biol.* 200, 839-850. 10.1083/jcb.201301120 23509072 PMC3601363

[JCS244376C6] Chevray P. M. and Nathans D. (1992). Protein interaction cloning in yeast: identification of mammalian proteins that react with the leucine zipper of Jun. *Proc. Natl. Acad. Sci. USA* 89, 5789-5793. 10.1073/pnas.89.13.5789 1631061 PMC402103

[JCS244376C7] Cohen Y. and Schuldiner M. (2011). Advanced Methods for High-Throughput Microscopy Screening of Genetically Modified Yeast Libraries in *Network Biology (Methods in Molecular Biology)*, Vol. 781 ( Cagney G. and Emili A.), pp. 127-159. Humana Press 10.1007/978-1-61779-276-2_8 21877281

[JCS244376C8] Cramer J., Effelsberg D., Girzalsky W. and Erdmann R. (2015). Isolation of Peroxisomes from Yeast. *Cold Spring Harb. Protoc.* 2015, pdb.top074500 10.1101/pdb.top074500 26330630

[JCS244376C9] DeLoache W. C., Russ Z. N. and Dueber J. E. (2016). Towards repurposing the yeast peroxisome for compartmentalizing heterologous metabolic pathways. *Nat. Commun.* 7, 11152 10.1038/ncomms11152 27025684 PMC5476825

[JCS244376C10] Effelsberg D., Cruz-Zaragoza L. D., Tonillo J., Schliebs W. and Erdmann R. (2015). Role of Pex21p for Piggyback Import of Gpd1p and Pnc1p into Peroxisomes of Saccharomyces cerevisiae. *J. Biol. Chem.* 290, 25333-25342. 10.1074/jbc.M115.653451 26276932 PMC4646183

[JCS244376C11] Effelsberg D., Cruz-Zaragoza L. D., Schliebs W. and Erdmann R. (2016). Pex9p is a new yeast peroxisomal import receptor for PTS1-containing proteins. *J. Cell Sci.* 129, 4057-4066. 10.1242/jcs.195271 27678487

[JCS244376C12] Erdmann R. and Blobel G. (1995). Giant peroxisomes in oleic acid-induced Saccharomyces cerevisiae lacking the peroxisomal membrane protein Pmp27p. *J. Cell Biol.* 128, 509-523. 10.1083/jcb.128.4.509 7860627 PMC2199900

[JCS244376C13] Erdmann R. and Kunau W.-H. (1994). Purification and immunolocalization of the peroxisomal 3-oxoacyl-CoA thiolase from Saccharomyces cerevisiae. *Yeast* 10, 1173-1182. 10.1002/yea.320100905 7754706

[JCS244376C14] Gabay-Maskit S., Schuldiner M. and Zalckvar E. (2018). Validation of a yeast malate dehydrogenase 2 (Mdh2) antibody tested for use in western blots. *F1000Res* 7, 130 10.12688/f1000research.13396.2 29568493 PMC5840644

[JCS244376C15] Götte K., Girzalsky W., Linkert M., Baumgart E., Kammerer S., Kunau W.-H. and Erdmann R. (1998). Pex19p, a farnesylated protein essential for peroxisome biogenesis. *Mol. Cell. Biol.* 18, 616-628. 10.1128/MCB.18.1.616 9418908 PMC121529

[JCS244376C16] Goward C. R. and Nicholls D. J. (1994). Malate dehydrogenase: a model for structure, evolution, and catalysis. *Protein Sci.* 3, 1883-1888. 10.1002/pro.5560031027 7849603 PMC2142602

[JCS244376C17] Harju S., Fedosyuk H. and Peterson K. R. (2004). Rapid isolation of yeast genomic DNA: Bust n’ Grab. *BMC Biotechnol.* 4, 8 10.1186/1472-6750-4-8 15102338 PMC406510

[JCS244376C18] Hofhuis J., Schueren F., Nötzel C., Lingner T., Gärtner J., Jahn O. and Thoms S. (2016). The functional readthrough extension of malate dehydrogenase reveals a modification of the genetic code. *Open Biol* 6, 160246 10.1098/rsob.160246 27881739 PMC5133446

[JCS244376C19] Huh W.-K., Falvo J. V., Gerke L. C., Carroll A. S., Howson R. W., Weissman J. S. and O'Shea E. K. (2003). Global analysis of protein localization in budding yeast. *Nature* 425, 686-691. 10.1038/nature02026 14562095

[JCS244376C20] James P., Halladay J. and Craig E. A. (1996). Genomic libraries and a host strain designed for highly efficient two-hybrid selection in yeast. *Genetics* 144, 1425-1436. 8978031 10.1093/genetics/144.4.1425 PMC1207695

[JCS244376C21] Kabran P., Rossignol T., Gaillardin C., Nicaud J.-M. and Neuveglise C. (2012). Alternative splicing regulates targeting of malate dehydrogenase in Yarrowia lipolytica. *DNA Res.* 19, 231-244. 10.1093/dnares/dss007 22368181 PMC3372373

[JCS244376C22] Kal A. J., van Zonneveld A. J., Benes V., van den Berg M., Koerkamp M. G., Albermann K., Strack N., Ruijter J. M., Richter A., Dujon B. et al. (1999). Dynamics of gene expression revealed by comparison of serial analysis of gene expression transcript profiles from yeast grown on two different carbon sources. *Mol. Biol. Cell* 10, 1859-1872. 10.1091/mbc.10.6.1859 10359602 PMC25383

[JCS244376C23] Kerssen D., Hambruch E., Klaas W., Platta H. W., de Kruijff B., Erdmann R., Kunau W.-H. and Schliebs W. (2006). Membrane association of the cycling peroxisome import receptor Pex5p. *J. Biol. Chem.* 281, 27003-27015. 10.1074/jbc.M509257200 16849337

[JCS244376C24] Kumar S., Singh R., Williams C. P. and van der Klei I. J. (2016). Stress exposure results in increased peroxisomal levels of yeast Pnc1 and Gpd1, which are imported via a piggy-backing mechanism. *Biochim. Biophys. Acta Mol. Cell Res.* 1863, 148-156. 10.1016/j.bbamcr.2015.10.017 26516056

[JCS244376C25] Kunze M., Pracharoenwattana I., Smith S. M. and Hartig A. (2006). A central role for the peroxisomal membrane in glyoxylate cycle function. *Biochim. Biophys. Acta Mol. Cell Res.* 1763, 1441-1452. 10.1016/j.bbamcr.2006.09.009 17055076

[JCS244376C26] McAlister-Henn L., Steffan J. S., Minard K. I. and Anderson S. L. (1995). Expression and function of a mislocalized form of peroxisomal malate dehydrogenase (MDH3) in yeast. *J. Biol. Chem.* 270, 21220-21225. 10.1074/jbc.270.36.21220 7673155

[JCS244376C27] McCammon M. T., Veenhuis M., Trapp S. B. and Goodman J. M. (1990). Association of glyoxylate and beta-oxidation enzymes with peroxisomes of Saccharomyces cerevisiae. *J. Bacteriol.* 172, 5816-5827. 10.1128/JB.172.10.5816-5827.1990 2211514 PMC526899

[JCS244376C28] McNew J. A. and Goodman J. M. (1994). An oligomeric protein is imported into peroxisomes in vivo. *J. Cell Biol.* 127, 1245-1257. 10.1083/jcb.127.5.1245 7962087 PMC2120261

[JCS244376C29] Metallo C. M. and Vander Heiden M. G. (2013). Understanding metabolic regulation and its influence on cell physiology. *Mol. Cell* 49, 388-398. 10.1016/j.molcel.2013.01.018 23395269 PMC3569837

[JCS244376C30] Minárik P., Tomaásková N., Kollárová M. and Antalík M. (2002). Malate Dehydrogenases - Structure and function. *Gen. Physiol. Biophys.* 21, 257-265. 12537350

[JCS244376C31] Pettersen E. F., Goddard T. D., Huang C. C., Couch G. S., Greenblatt D. M., Meng E. C. and Ferrin T. E. (2004). UCSF Chimera–a visualization system for exploratory research and analysis. *J. Comput. Chem.* 25, 1605-1612. 10.1002/jcc.20084 15264254

[JCS244376C32] Šali A. and Blundell T. L. (1993). Comparative protein modelling by satisfaction of spatial restraints. *J. Mol. Biol.* 234, 779-815. 10.1006/jmbi.1993.1626 8254673

[JCS244376C33] Saryi N. A. A., Hutchinson J. D., Al-Hejjaj M. Y., Sedelnikova S., Baker P. and Hettema E. H. (2017). Pnc1 piggy-back import into peroxisomes relies on Gpd1 homodimerisation. *Sci. Rep.* 7, 42579 10.1038/srep42579 28209961 PMC5314374

[JCS244376C34] Schäfer A., Kerssen D., Veenhuis M., Kunau W.-H. and Schliebs W. (2004). Functional similarity between the peroxisomal PTS2 receptor binding protein Pex18p and the N-terminal half of the PTS1 receptor Pex5p. *Mol. Cell. Biol.* 24, 8895-8906. 10.1128/MCB.24.20.8895-8906.2004 15456864 PMC517879

[JCS244376C35] Schueren F., Lingner T., George R., Hofhuis J., Dickel C., Gärtner J. and Thoms S. (2014). Peroxisomal lactate dehydrogenase is generated by translational readthrough in mammals. *eLife* 3, e03640-e03640 10.7554/eLife.03640 PMC4359377 25247702

[JCS244376C36] Steffan J. S. and McAlister-Henn L. (1992). Isolation and characterization of the yeast gene encoding the MDH3 isozyme of malate dehydrogenase. *J. Biol. Chem.* 267, 24708-24715. 1447211

[JCS244376C37] Tower R. J., Fagarasanu A., Aitchison J. D. and Rachubinski R. A. (2011). The peroxin Pex34p functions with the Pex11 family of peroxisomal divisional proteins to regulate the peroxisome population in yeast. *Mol. Biol. Cell* 22, 1727-1738. 10.1091/mbc.e11-01-0084 21441307 PMC3093324

[JCS244376C38] Van Der Spoel D., Lindahl E., Hess B., Groenhof G., Mark A. E. and Berendsen H. J. C. (2005). GROMACS: fast, flexible, and free. *J. Comput. Chem.* 26, 1701-1718. 10.1002/jcc.20291 16211538

[JCS244376C39] Wanders R. J., Waterham H. R. and Ferdinandusse S. (2015). Metabolic interplay between peroxisomes and other subcellular organelles including mitochondria and the endoplasmic reticulum. *Front. Cell Dev. Biol.* 3, 83 10.3389/fcell.2015.00083 26858947 PMC4729952

[JCS244376C40] Waterham H. R., Ferdinandusse S. and Wanders R. J. A. (2016). Human disorders of peroxisome metabolism and biogenesis. *Biochim. Biophys. Acta Mol. Cell Res.* 1863, 922-933. 10.1016/j.bbamcr.2015.11.015 26611709

[JCS244376C41] Weill U., Yofe I., Sass E., Stynen B., Davidi D., Natarajan J., Ben-Menachem R., Avihou Z., Goldman O., Harpaz N. et al. (2018). Genome-wide SWAp-Tag yeast libraries for proteome exploration. *Nat. Methods* 15, 617-622. 10.1038/s41592-018-0044-9 29988094 PMC6076999

[JCS244376C42] Yan Tong A. H. and Boone C. (2005). Synthetic genetic array analysis in Saccharomyces cerevisae. *Methods Mol. Biol.* 313, 171-191. 10.1385/1-59259-958-3:171 16118434

[JCS244376C43] Yang X., Purdue P. E. and Lazarow P. B. (2001). Eci1p uses a PTS1 to enter peroxisomes: either its own or that of a partner, Dci1p. *Eur. J. Cell Biol.* 80, 126-138. 10.1078/0171-9335-00144 11302517

[JCS244376C44] Yifrach E., Chuartzman S. G., Dahan N., Maskit S., Zada L., Weill U., Yofe I., Olender T., Schuldiner M. and Zalckvar E. (2016). Characterization of proteome dynamics during growth in oleate reveals a new peroxisome-targeting receptor. *J. Cell Sci.* 129, 4067-4075. 10.1242/jcs.195255 27663510 PMC6275125

[JCS244376C45] Yifrach E., Fischer S., Oeljeklaus S., Schuldiner M., Zalckvar E. and Warscheid B. (2018). Defining the mammalian peroxisomal proteome. *Subcell. Biochem.* 89, 47-66. 10.1007/978-981-13-2233-4_2 30378018

[JCS244376C46] Yofe I. and Schuldiner M. (2014). Primers-4-Yeast: a comprehensive web tool for planning primers for Saccharomyces cerevisiae. *Yeast* 31, 77-80. 10.1002/yea.2998 24408512

[JCS244376C47] Yofe I., Weill U., Meurer M., Chuartzman S., Zalckvar E., Goldman O., Ben-Dor S., Schütze C., Wiedemann N., Knop M. et al. (2016). One library to make them all: streamlining the creation of yeast libraries via a SWAp-Tag strategy. *Nat. Methods* 13, 371-378. 10.1038/nmeth.3795 26928762 PMC4869835

