# Supplementary material for: A piggybacking mechanism enables peroxisomal localization of the glyoxylate cycle enzyme Mdh2 in yeast
Source: J Cell Sci. 2020 Dec 17;133(24):jcs244376. doi: 10.1242/jcs.244376 (PMC7758625; doi:10.1242/jcs.244376)
Supplement: Supplementary information [file joces-133-244376-s1.pdf]

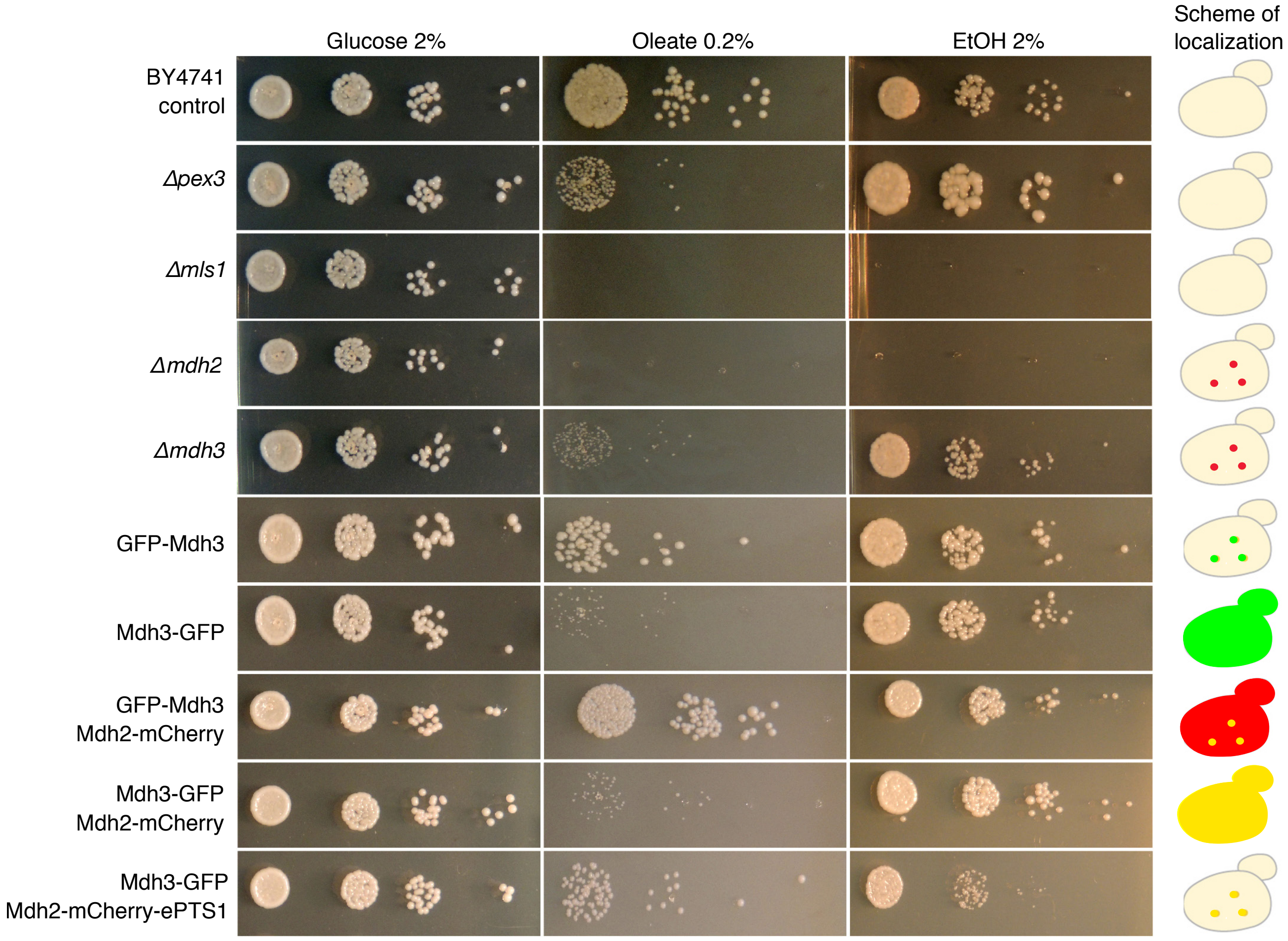

Journal of Cell Science • Supplementary information

Figure S1. Cells can consume oleate when either Mdh3 or Mdh2 contains a PTS1 signal. Left – Serial dilution growth assay on glucose, oleate and ethanol (EtOH). Right - A scheme of the protein localization as observed microscopically. Only when the PTS1 domain of Mdh3 was exposed (N' GFP tag, GFP-Mdh3), Mdh3 and Mdh2 were localized to peroxisomes and cells could grow on oleate. When an enhanced PTS1 (ePTS1) was added to Mdh2 (Mdh3-GFP; Mdh2-mCherry-ePTS1), the two enzymes were localized to peroxisomes and cells could consume oleate. However, the ePTS1 strain had a growth defect when grown on EtOH indicating that adding the ePTS1 to Mdh2 reduced its amount in the cytosol and is not any more available for the glyoxylate cycle that takes place in the cytosol upon growth on EtOH. The reduced growth ability on oleate implies that a cytosolic fraction of Mdh2 is also required for NAD+ recycling during β oxidation of fatty acids. In the *Δ mdh2* and *Δ mdh3* strains, Pex3 was C' tagged with mCherry to enable peroxisome visualization.

Journal of Cell Science • Supplementary information

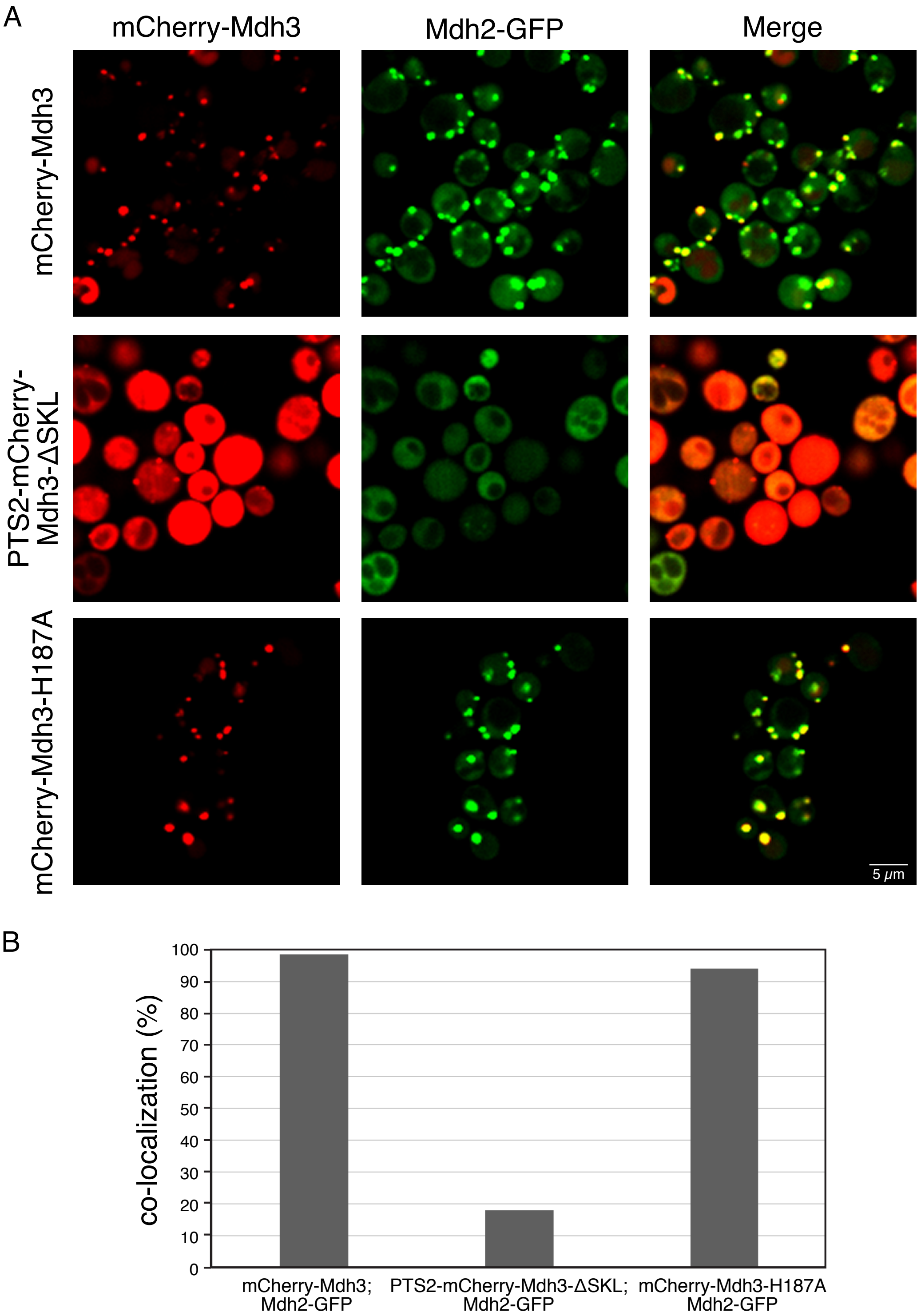

Journal of Cell Science • Supplementary information

Figure S2. Mdh2 piggybacking on Mdh3 is not supported by the Pex7 targeting machinery. (A)"GFP-Mdh2 Δ*mdh3* strain was transformed with a centromeric (CEN) plasmid coding for"mCherry-tagged Mdh3 (mCherry-Mdh3), the same protein but with its PTS1 deleted and a PTS2 added on its N' (PTS2-mCherry-Mdh3ΔSKL), or the same protein mutated at its enzymatic active site (mCherry-Mdh3-H187A). While targeting of PTS2-mCherry-Mdh3ΔSKL was inefficient and only a small fraction of Mdh3 localized to peroxisomes, we could see that even when Mdh3 was targeted properly, Mdh2 could not be detected in puncta in this strain implying that the piggybacking is specific to Pex5 targeting. (B) Percentage of Cherry-Mdh3 puncta that co-localized to Mdh2-GFP puncta in cells expressing different Mdh3 plasmids (as indicated above). Data represent average percentage from two repeats, 200 puncta per strain in total.

Supplementary Tables

Table S1. List of yeast strains included in the peroxisome deletion library

Click here to Download Table S1

Table S2. List of yeast strains used in this study

Click here to Download Table S2

Table S3. List of plasmids used in this study

Click here to Download Table S3

Table S4. List of primers used in this study

Click here to Download Table S4

Journal of Cell Science • Supplementary information

Supplementary References

(Goldstein and McCusker, 1999; James et al., 1996; Janke et al., 2004; Longtine et al., 1998)

Goldstein, A.L., and McCusker, J.H. (1999). Three new dominant drug resistance cassettes for gene disruption in Saccharomyces cerevisiae. Yeast 15, 1541-1553.

James, P., Halladay, J., and Craig, E.A. (1996). Genomic libraries and a host strain designed for highly efficient two-hybrid selection in yeast. Genetics 144, 1425-1436.

Janke, C., Magiera, M.M., Rathfelder, N., Taxis, C., Reber, S., Maekawa, H., Moreno-Borchart, A., Doenges, G., Schwob, E., Schiebel, E., et al. (2004). A versatile toolbox for PCR-based tagging of yeast genes: new fluorescent proteins, more markers and promoter substitution cassettes. Yeast 21, 947-962.

Longtine, M.S., McKenzie, A., 3rd, Demarini, D.J., Shah, N.G., Wach, A., Brachat, A., Philippsen, P., and Pringle, J.R. (1998). Additional modules for versatile and economical PCR-based gene deletion and modification in Saccharomyces cerevisiae. Yeast 14, 953-961.
